# Correction: Scabies prevalence and its associated factors among prisoners in southern Ethiopia: An institution-based analytical cross-sectional study

**DOI:** 10.1371/journal.pntd.0013220

**Published:** 2025-06-25

**Authors:** Efa Ambaw Bogino, Beshada Zerfu Woldegeorgis, Lantesil Wondewosen, Blen Kassahun Dessu, Mohammed Suleiman Obsa, Lolemo Kelbiso Hanfore, Teketel Ermias Geltore, Woldu Kidane, Abraham Getachew Kelbore

The seventh author’s name is spelled incorrectly. The correct name is: Teketel Ermias Geltore.
