# Supplementary material for: Scabies prevalence and its associated factors among prisoners in southern Ethiopia: An institution-based analytical cross-sectional study
Source: PLoS Negl Trop Dis. 2023 Dec 22;17(12):e0011826. doi: 10.1371/journal.pntd.0011826 (PMC10773954; doi:10.1371/journal.pntd.0011826)
Supplement: Attachment — Submitted filename: Response to Reviewers.docx [file pntd.0011826.s001.docx]

**Response to reviewers**

I. **General note for academic editor regarding the overall progress**

First and foremost, we would like to thank the academic editor for allowing us sufficient time to revise and address all the reviewers' concerns and journal requirements. The reviewer's comments were critically evaluated, and necessary corrections or amendments were made. The manuscript has been closely examined for typographical errors; Lastly, for comparison, we provided a response to the reviewer’s comment under each concern. We hope the academic editor and expert reviewers receive a significantly improved manuscript. Thank you incredibly much.!

Detailed list of our responses to all reviewer’s comment and description of the changes made in the manuscript was explained.

Two versions of revised manuscript, one tracked change and other clean version were uploaded.

Finally, all authors agreed on the edited version of the manuscript declaring there is no conflict of interest.

**II.	Author’s response for reviewer #1**

Greetings, dear reviewer #1: we are writing to express our gratitude and appreciation for reviewing our manuscript, "**Scabies prevalence and its associated factors among prisoners in Southern Ethiopia**." We thoroughly addressed specific points made and uploaded a marked-up copy (revised manuscript with tracked changes), and a clean copy as “manuscript” file. At this point, again, we would like to thank you for taking the time to provide invaluable comments to enrich the contents and make them more palatable to the scientific community in general.

**INTRODUCTION**

**Reviewer comment 1:** Probably better: Start a new sentence. The mites cause health problems (instead of Pest problems) in resource-scarce countries. I would say that often leads to serious skin infections (Without “often” it is too absolute) It will be nice to mention for each study whether it is done in communities or in prisons.

**-Author’s response**: The reviewer’s suggestion is appropriate. We incorporated the comments into the revised /marked manuscript. Thank you very much. Page # 3, line 76 and 77

**METHODS**

**Reviewer’s comment 2:** In measurement I suppose you looked for the typical lesions in order to diagnose. Case definition could be better placed before measurement. Then you do not have to repeat in this section. The first mention of the genital area should be deleted.

**-Authors’ response:** Thank you for your suggestion; in the revised document we placed the case definition before the measurement section. Furthermore, the duplicated word i.e “genital lesion” was deleted from the initial sentence. Page #5, line 137- 142

**RESULT**

**Reviewer’s comment 3:** 4 refused. You did not replace them to get your 422?

**-Authors’ response:** Thank you for your question**,** unfortunately our survey had less than 1% non-response rate. Moreover, we assumed that the characteristics of the non-respondents (those 4 refused persons) are similar with individuals who were surveyed**.** As a result, we did not expect it to cause nonresponse bias. Page #6, line 183

**Reviewer’s comment 4:** Table 1. In the frequency of gender is written a percentage of what?

**-Authors’ response:** Regarding the gender, it’s an edition problem. Now the percentage has been removed from the frequency column. Page #6

**Reviewer’s comment 5:** There was one room with 326 inmates.  Did you look separate to this room for interest?  I suppose this room was cleaned daily. Did this fit with the responses?

**-Authors’ response:** We haven’t seen separately for the room which has 326 inmates because we took our study subjects from sample frame (register) of the prisoners, not from individual rooms as well the room assignment was changed upon release and entry of inmates. Therefore, on this study subgroup analysis was not done. And as you suppose the prisoners reported that the room was cleaned on daily base.

**Reviewer’s comment 6**: Table 3, Daily room cleaning claimed by 325. How many rooms are there in total?

**-Authors Response**: Yes, your point of view is correct. Daily room cleaning claimed by 352 prison inmates. Off these, 325 were not infested with scabies. Totally there were 6 rooms in the prison. Page #7, line 194 and Page 9 Table: 2

**Reviewer’s Comment 7**: Is there a relationship between sex in the last two months and the duration of the stay in prison?
Is there one factor related to higher educated having more scabies? (More frequently sex?) Maybe just in this prison.
Interesting the indication that more frequent cloth changes lead to more scabies. How are the bedclothes cleaned? Are they shared?

**-Author’s Response**: Your comment is well accepted, and we understand your perspective justifications.

Just in this study educational status has no significant association with scabies. In addition the number of male prisoner inmates were higher than female.

Frequent changes of clothing have preventive effect on scabies infestation. But unfortunately, on this study we found that frequent personnel change of clothing by itself doesn’t prevent scabies infestation among prisoners. This could be justified because prisoners share their night clothes and, they have intimate physical contact in the room, which puts them at risk of contracting scabies from an infected index patient even though they change clothes daily.

**Reviewer’s comment 8:** I wondered what is meant by room cleaning. Scabies do not survive on the floor. Do they sleep always in the same bed?

**-Author’s response**: For this study, Cleaning a room is as simple as sweeping away the dust. We agree with the reviewer on room cleanliness suggested comment; nonetheless, room cleaning alone does not prevent scabies infestation unless it is supplemented by restrictions on cloth sharing, limiting body contact with inmates, and other scabies prevention precautions.

**Reviewer’s comment 9**: Do bedclothes changing belong to daily room cleaning?

**-Author’s Answer:** NO

III. **Authors response for reviewer #2**

Greetings, dear Reviewer 2: I'm writing to express my gratitude of appreciation for general comment and taking the time to review our manuscript, "Scabies prevalence and associated factors among prisoners in southern Ethiopia” We thoroughly addressed specific points made and uploaded a marked-up copy (revised manuscript with tracked changes) as a "Revised Article with Changes Highlighted” file, and a clean copy as “manuscript” file. At this point, we would like to thank you for taking the time to provide invaluable comments to enrich the contents and make them more palatable to the scientific community in general.

**Reviewer’s Comment 1**: There are some formal errors that should be corrected.

**-Author’s response:** Thank you for your comment. We thoroughly seen the document and formal errors were corrected(including typos, grammar, spelling were corrected)

**Reviewer’s Comment 2:** abbreviations LIC's, MIC's need to be explained.

**-Author’s response:** Those abbreviations (LIC’s and MIC’s) were explained in the document. ( Page #2, line 88)

**Reviewer’s Comment 3**. page 7, were patients only informed verbally, no written informed consent?

**-Author’s Response**: Thank you for your in-depth review and acknowledged your comment. Regarding verbal consent, it was mistaken while writing the manuscript. All prison inmates who have been enrolled on this study were consented before interview in written format. (page #6, line 177)

**Reviewer’s Comment 4**: page 8, were the mites or feces or eggs not detected by microscopical or dermatoscopical investigation to make the exact diagnosis?

**Author’s response**: Thank you for your question. As you know here in developing countries there is shortage of resources. For the exact diagnosis of scabies, we didn’t get the equipment that is why we used IACS criteria for diagnosis. So, this could be the limitation of the study.(page #5, line 150-152)
